# Follistatin-like 1: a novel biomarker with a potential link to obstructive sleep apnea severity and treatment efficacy

**DOI:** 10.3389/fendo.2025.1690401

**Published:** 2025-12-11

**Authors:** Abdulmohsen Alterki, Mohamed Abu-Farha, Eman Al Shawaf, Aldana Alrashidi, Nouf Alsuhail, Irina Al-Khairi, Preethi Cherian, Dhanya Madhu, Devarajan Sriraman, Mahmoud Ebrahim, Mohammed Alterki, Saadoun Bin-Hasan, Fahd Al-Mulla, Jehad Abubaker

**Affiliations:** 1 Consultant Otolaryngology, Head & Neck Surgery, Department Otolaryngology, Head & Neck Surgery, Zain & Al Sabah Hospitals, Kuwait, Kuwait; 2 Chairman of Otolaryngology, Head & Neck Surgery Faculty, Kuwait Board of Post Graduate Training Program, Kuwait Institute for Medical Specializations (KIMS), Kuwait, Kuwait; 3 Research Sector, Dasman Diabetes Institute, Dasman, Kuwait; 4 Department of Biochemistry and Molecular Biology, Dasman Diabetes Institute, Dasman, Kuwait; 5 Special Service Facility Department, Dasman Diabetes Institute, Dasman, Kuwait; 6 Department of Otolaryngology, McGill University, Montreal, QC, Canada; 7 Department of General Surgery, Farwaniya Hospital, Ministry of Health, Kuwait, Kuwait; 8 Director of Sleep Medicine and staff pediatric respirologist, Farwaniya Hospital, Sabah Al Nasser, Kuwait; 9 Translational Research Department, Dasman Diabetes Institute, Dasman, Kuwait

**Keywords:** obstructive sleep apnea, polysomnography, multilevel sleep surgery, hypoxia, follistatin-like 1, biomarker

## Abstract

**Introduction:**

Obstructive sleep apnea (OSA) is a common sleep disorder characterized by intermittent hypoxia, systemic inflammation, and metabolic dysfunction. Current diagnostic standards rely on polysomnography (PSG), which is limited by cost and accessibility. The identification of a sensitive and specific biomarker has the potential to aid both diagnosis and treatment monitoring. Follistatin-like 1 (FSTL1) has been implicated in inflammatory pathways; however, its role in OSA remains largely unexplored.

**Materials and Methods:**

In this study, we aimed to explore changes in circulating FSTL1 levels in individuals with OSA to assess alterations following multilevel sleep surgery (MLS). We also evaluated its association with various metabolic and hypoxia-related markers, including Orexin-A, TNF-α, and IGFBP4. Our study was conducted at Dasman Diabetes Institute (DDI) in Kuwait through a cohort of 164 individuals, comprising 124 patients with OSA and 40 participants as non-OSA controls. Participants with OSA underwent MLS as a corrective intervention. A Type I polysomnography (PSG) test was performed in a level 1 sleep laboratory to diagnose sleep apnea. The apnea-hypopnea index (AHI) was measured at baseline and 3 months post-surgery to evaluate improvement in their condition.

**Results:**

Circulating FSTL1 levels were significantly lower in individuals with OSA (10,245.53 ± 174.94; p < 0.001) compared to the control group (13,783.33 ± 688.69), with levels restored following surgery. Our data presented an inverse association between FSTL1 and AHI (p < 0.001), highlighting its potential use in reflecting OSA severity. Additionally, FSTL1 levels showed a significant negative correlation with the hypoxia-related marker IGFBP4 in OSA participants (r = –0.440; p = 0.005), suggesting a potential link to hypoxic regulation. FSTL1 levels increased significantly (p = 0.041) following MLS, coinciding with improvements in AHI and indicating remission of OSA. Further, the receiver operating curve (ROC) analysis emphasized a potential role for FSTL1 as a biomarker with predictive qualities for OSA, showing moderate diagnostic accuracy (AUC 0.73, 95% CI: 0.64–0.83, p < 0.001; 8819.09; sensitivity of 86.4%, specificity of 76.2%).

**Conclusion:**

FSTL1 demonstrates potential as a valuable biomarker that can aid current diagnostic tools for OSA and help evaluate treatment efficacy; however, additional research is warranted to confirm its clinical applicability and explore its therapeutic potential.

## 1 Introduction

Obstructive sleep apnea (OSA) is a common sleep disorder characterized by recurrent episodes of upper airway obstruction during sleep, resulting in intermittent hypoxic events, lowered oxygen levels, and disturbed sleep (1). A key pathogenic driver is intermittent hypoxia, which triggers oxidative stress and activates transcription factors such as hypoxia-inducible factor-1α (HIF-1α) and nuclear factor kappa B (NF-κB). These pathways promote systemic inflammation, infiltration of proinflammatory M1 macrophages, vascular injury, and endothelial dysfunction, ultimately contributing to cardiometabolic complications (1–3). More recently, inflammasome signaling has been highlighted as a key mediator linking hypoxia to tissue injury and immune activation, whereby this multiprotein complex promotes the release of pro-inflammatory cytokines such as IL-18 and IL-1β, driving systemic inflammation and contributing to the neurological and cardiovascular complications observed in OSA (4). OSA is particularly prevalent among individuals with risk factors such as obesity, advancing age, male gender, and family history, and is caused by the relaxation of pharyngeal muscles, which leads to partial or complete airway obstruction (1). OSA symptoms are manifested as episodes of choking or gasping during sleep, excessive daytime somnolence, concentration difficulty, loud snoring, and morning headaches (1). Persistent and untreated OSA leads to a reduction in the oxygen levels and induction of oxidative stress, which substantially contributes to the development of hypertension, cardiovascular disease, and an increased stroke risk (2, 3).

The primary OSA detection and diagnosis method is an extensive sleep test, polysomnography (PSG), that comprehensively monitors multiple physiological parameters during sleep, including brain activity, eye movements, muscle activity, heart rhythms, respiratory function, oxygen saturation, and chest movement (5). PSG provides critical insights into sleep disturbances and the severity classification of OSA. In parallel, research has identified several novel biomarkers with potential diagnostic and prognostic relevance in OSA, such as ADAM29, FLRT2 and SLC18A3, Endocan, YKL-40, IL-6 and Vimentin levels (6). These include inflammatory cytokines (e.g., IL-6, TNF-α, CRP), circulating proteins involved in vascular and metabolic regulation, and genetic and epigenetic variants associated with hypoxia response and metabolic pathways (7). Additionally, a growing body of evidence implicates microRNAs in the regulation of inflammation, oxidative stress, and insulin signaling in OSA (8). Together, these findings provide a rich framework for biomarker discovery and support the evaluation of novel candidates in OSA pathophysiology.

OSA management includes various treatment options to maintain airway patency and reduce apneic events. This includes strategies for mild–to–moderate OSA, like lifestyle modifications, positional therapy, mandibular advancement devices (MADs), and increased physical activity (9). Nevertheless, advanced and severe OSA cases are treated with methods that restore normal oxygenation during sleep by preventing airway blockage or collapse, such as continuous positive airway pressure (CPAP) and multilevel sleep surgery (MLS) that restore normal oxygenation during sleep by preventing airway blockage or collapse (9). MLS is a comprehensive alternative approach that addresses multiple sites of obstruction in a single individualized procedure (5, 9). These procedures aim to eliminate intermittent hypoxia, reduce oxidative stress and systemic inflammation, and improve cardiovascular outcomes.

Examining the molecular mechanisms affected by chronic intermittent hypoxia, oxidative stress, and systemic inflammation has directed OSA research toward molecular pathways regulating these processes. Follistatin-like 1 (FSTL1) is a secreted glycoprotein involved in inflammation, cardiovascular regulation, and cellular stress responses (10). Initial studies identified FSTL1 as a transforming growth factor-beta (TGF-β)-inducible protein that plays a role in vascularization, immune modulation, and cellular adaptation to hypoxia (10). In a recent study, FSTL1 was downregulated in the lung tissues of mice exposed to intermittent hypoxia (IH) and inoculated with melanoma cells, suggesting a possible involvement of FSTL1 in IH-induced tumor lung metastasis (11). FSTL1-deficient mice exhibited increased susceptibility to lung metastases, characterized by increased oxidative stress, inflammation, and tumor progression (11). This occurred with a significant upregulation in hypoxia markers, hypoxia-inducible factor 1-alpha (HIF-1α) and vascular endothelial growth factor (VEGF), along with pro-inflammatory cytokines such as tumor necrosis factor-alpha (TNF-α) and interleukin-6 (IL-6) (11).

Considering that chronic intermittent hypoxia, a main feature of OSA, suppresses FSTL1 expression, this suggests a potential protective role for FSTL1 in OSA patients by regulating inflammation and oxidative stress; however, this role has not been studied. Investigating how FSTL1 levels fluctuate in response to OSA severity and treatment interventions, such as MLS, could provide valuable insights into its function as a biomarker. Understanding these mechanisms may pave the way for novel tools to aid therapeutic strategies in mitigating the systemic complications associated with OSA. Furthermore, assessing its diagnostic and prognostic sensitivity may help determine its utility for detecting disease, monitoring progression, and evaluating treatment response.

## 2 Materials and methods

### 2.1 Study population and sample collection

This cohort study was undertaken at DDI in Kuwait and conducted in accordance with the Declaration of Helsinki, following approval from the institute’s ethics committee. Informed written consent was obtained from all participants upon enrolment. A total of 164 individuals joined the study, of whom 124 were diagnosed with OSA and 40 were control participants. Baseline demographic and clinical data, encompassing the apnea-hypopnea index (AHI), were collected for all participants. Subsequent measurements were obtained for patients undergoing MLS to evaluate treatment efficacy.

Participants were recruited from the sleep clinic at DDI following the study’s criteria. Eligible participants were adults (18–65 years) referred for evaluation of suspected obstructive sleep apnea (OSA). Exclusion criteria included a prior history of cardiovascular disease, diabetes mellitus, active cancer, chronic kidney disease, acute inflammatory or infectious conditions, or obesity with body mass index (BMI) >35; other BMI ranges were included, and BMI was entered as a covariate, as these conditions also contraindicate OSA surgery. Obesity per se was not an exclusion criterion due to its strong association with OSA and its relevance to the study objectives. BMI was recorded for all participants and included as a covariate in statistical analyses to adjust for its potential confounding effect. Fasting blood samples were collected from all participants at baseline in Vacutainer EDTA tubes containing aprotinin. The collected samples were centrifuged at 400 x g for 10 minutes at room temperature to extract plasma that was aliquoted and stored at -80 °C for future analysis.

### 2.2 OSA assessment and surgery

The study population was diagnosed using the Type I PSG test in a level 1 sleep laboratory to determine the presence of sleep apnea, as previously described (12). This overnight sleep laboratory assessment records multiple biophysiological signals to provide a comprehensive evaluation of sleep architecture. Specifically, Type I PSG monitors blood oxygen saturation, respiratory effort and airflow, brain activity (EEG), eye movement (EOG), heart rhythm (ECG), and skeletal muscle activity (EMG). These measures are used to calculate the apnea–hypopnea index (AHI). The apnea index (AI) reflects the number of complete respiratory cessations lasting >10 seconds per hour, while the hypopnea index (HI) reflects partial airway obstructions per hour. Diagnosis of OSA was based on AHI scores, with >5 events/h considered abnormal. AHI severity thresholds were defined as: 5–15 events/h (mild), 15–30 events/h (moderate), and >30 events/h (severe) (13). Every surgery included tonsillectomy, complemented by additional corrections involving the nasal surgery and other procedures like palatal or base of tongue depending on the site of upper-airway obstruction, after performing drug induced sleep endoscopy (DISE) prior to surgery, and the multilevel surgery was done in a single stage by the same surgeon.

### 2.3 Quantification of circulating follistatin-like 1 levels

Levels of follistatin-like 1 (FSTL1) were quantified with a multiplex immunoassay (Luminex custom-made panel, cat #LXSAHM, R&D Systems, CA, USA) using the Bio-Plex Manager software version 6.0 (Bio-Rad, Hercules, CA, USA). For the standard curve fitting analysis, we employed a five-parameter logistic (5-PL) nonlinear regression model. The intra-assay coefficient of variation ranged from 1.2% - 3.8%, while the inter-assay coefficient of variation ranged from 2.8% - 5.2% ensuring data reproducibility.

### 2.4 Quantification of the levels of different circulating metabolic markers

Circulating levels of c-peptide and insulin were measured by ELISA, and assays were performed according to the kit instructions (Mercodia AB, Sylveniusgatan, Sweden; Cat# 10-1136–01 and Cat# 10-1113-01, respectively). Absorbance was determined for standards and samples at 450 nm using the Synergy HTx plate reader (BioTek, Agilent, CA, USA). The Concentration of the unknown samples was interpolated from the standard curve; all data were generated by Gen5 software. The intra-assay coefficient for these ELISA assays was 2.0%–5.0%, while the inter-assay coefficient was < 10%.

Levels of circulating IGFBP-4, TNF-α, and Leptin were quantified using a custom multiplex immunoassay (Luminex custom-made panel, cat #LXSAHM, R&D Systems, CA, USA). The results were calculated using the Bio-Plex Manager software version 6.0 (Bio-Rad, Hercules, CA, USA). For the standard curve fitting analysis, we employed a five-parameter logistic (5-PL) nonlinear regression model. The intra-assay coefficient of variation ranged from 1.2% - 3.8%, while the inter-assay coefficient of variation ranged from 2.8% - 5.2% ensuring data reproducibility.

### 2.5 Statistical analysis

Data were normalized using the inverse distribution function (IDF). Continuous variables are presented as mean ± standard error of the mean (SEM), and categorical variables are presented as frequencies and percentages. Between-group comparisons were performed using independent Student’s t-tests or Mann–Whitney U tests for continuous variables and χ² tests for categorical variables, while a paired T-test assessed within-group differences between baseline and the 3-month follow-up for only OSA group. Normality was assessed using the Shapiro–Wilk test, and homogeneity of variance was evaluated using Levene’s test. Associations between circulating FSTL1 and clinical parameters were examined using Pearson correlation coefficients. Multivariate linear regression analysis was conducted to identify independent predictors of FSTL1 levels. Variables included in the regression model were selected based on clinical relevance and univariate significance, with checks for multicollinearity. Detailed results of the BMI-stratified analyses are presented in **Supplementary Table 1**. Receiver operating characteristic (ROC) analysis was performed to evaluate the discriminatory ability of FSTL1 for moderate-to-severe OSA. All statistical analyses were conducted using SPSS for Windows, version 25.0 (IBM SPSS Inc., USA). A p-value of <0.05 was considered statistically significant. The primary endpoint was prespecified; secondary analyses were exploratory; no formal multiple-testing correction was applied.

#### 2.5.1 Sample power

*A priori* power analysis was performed to ensure adequacy of the study sample. For the primary endpoint (between-group differences in circulating FSTL1 levels between OSA and controls), assuming a two-tailed α=0.05, a moderate effect size (Cohen’s d=0.5), and 80% power, the minimum required size was 64 participants (32 per group). For 90% power, 86 participants were required. Our final cohort (OSA = 124; controls=40) exceeded these requirements, ensuring adequate sensitivity. The observed effect size was large (d=1.30), corresponding to >99.9% achieved power.

## 3 Results

### 3.1 Study cohort, baseline features

**Figure 1:**
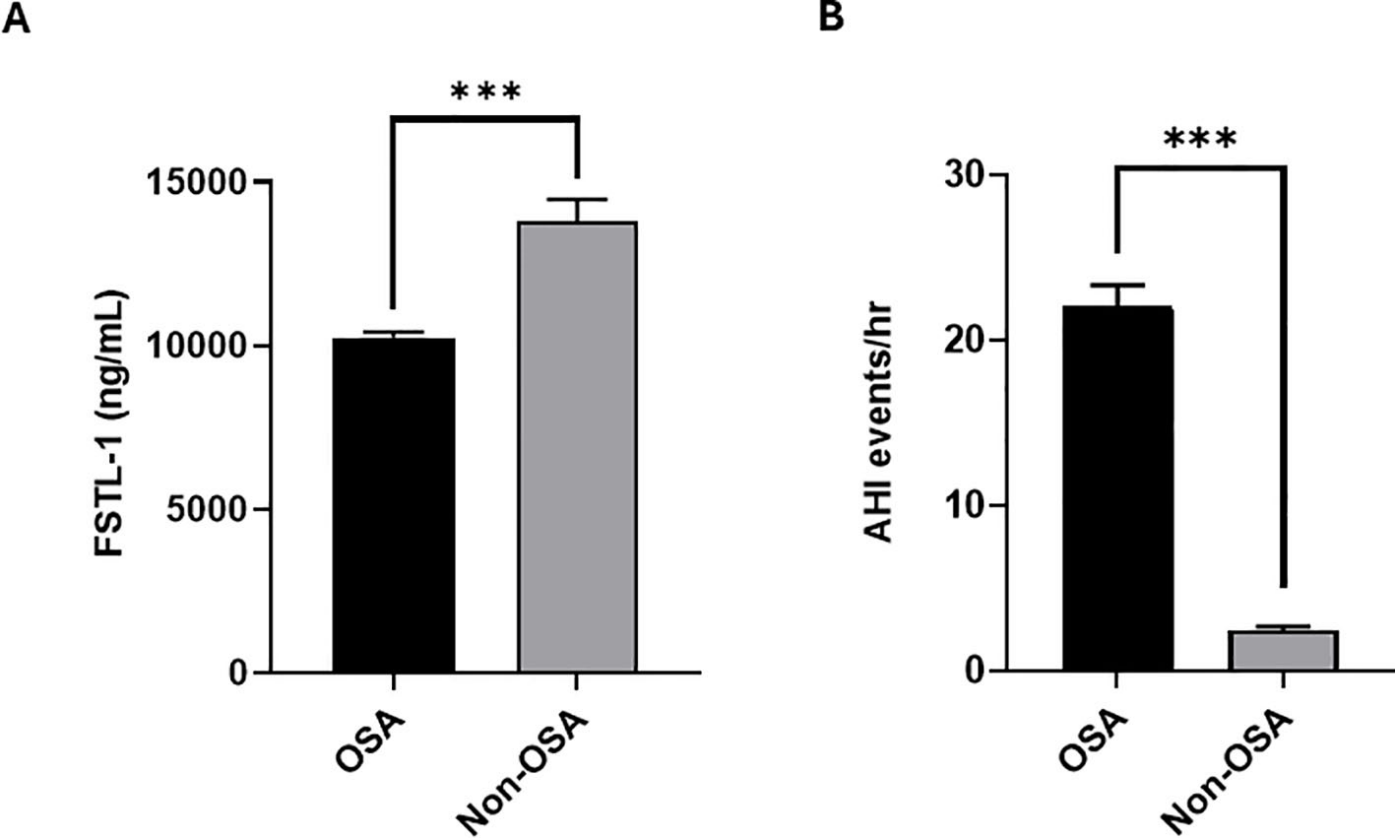
Circulating FSTL1 levels in OSA and control (CTRL) groups. **(A)** Baseline FSTL1 levels were significantly lower in individuals with OSA (10245.53 ± 174.94; p < 0.001, n=124) compared to the CTRL group (13783.33 ± 688.69, n=40), while AHI was significantly higher in the OSA group (22.06 ± 1.25; p < 0.001) compared to the non-OSA group (2.45 ± 0.22, **B**).

The current report presents a cohort of 164 participants, categorized according to OSA diagnosis into 124 with OSA and 40 CTRL (i.e., people with no OSA). Most participants were men, representing 84% of the study population (**Table 1**), compared to women who made up ~16% of the cohort. Notably, FSTL1 was significantly lower in people with OSA (<0.001, **Figure 1A**) than non-OSA individuals, reinforcing a possible link between OSA and FSTL1 dysregulation. Increased Apnea-hypopnea index (AHI ≥5) was used to identify participants with OSA (AHI = 22.06 ± 1.25 events/hour **Figure 1B**) while the CTRL showed significantly lower AHI index (AHI = 2.45 ± 0.22 events/hour). Moreover, participants with OSA had significantly higher Epworth Sleepiness Scale (ESS) scores (p < 0.001) compared to CTRL (**Table 1**). Additionally, various biochemical parameters and metabolic markers, including c-peptide (p = 0.006) and insulin (p = 0.025) were elevated in people with OSA, suggesting insulin resistance. Next, we stratified the cohort based on obesity and incorporated the corresponding clinical characteristics. The BMI-stratified analysis (**Supplementary Table 1**) revealed significant differences in ESS, OSA indices (AI, HI, AHI), HbA1c, C-peptide, insulin, IGFBP4, and leptin levels in the obese group, consistent with obesity being an independent contributor to OSA severity and metabolic dysregulation. Notably, FSTL1 levels were not influenced by obesity (obese: 11,045.46 ± 265.51 ng/mL vs. non-obese: 11,458.37 ± 618.22 ng/mL; p = 0.544), indicating that the associations between FSTL1 and OSA severity are obesity-independent. Sex-specific effects were not tested in our study and should be validated in larger, gender-balanced cohorts.

**Table 1:** Baseline clinical and biochemical characteristics of participants.

Variables	OSA (n=124) mean ± SEM	Non-OSA (n=40) mean ± SEM	p-value
Age (Years)	43.55 ± 1.00	44.75 ± 1.77	0.557
Gender (M/F)^#^	105/19	33/7	0.745
ESS	13.32 ± 1.36	4.00 ± 1.33	**<0.001**
Weight (Kg)	91.23 ± 1.61	82.68 ± 2.50	**0.005**
Height (cm)	171.23 ± 0.79	170.99 ± 1.53	0.889
Body Mass Index (kg/m2)	31.04 ± 0.46	27.94 ± 0.68	**<0.001**
Pulse	74.94 ± 1.01	74.15 ± 1.75	0.698
SBP (mmHg)	126.21 ± 1.21	123.74 ± 1.85	0.270
DBP (mmHg)	73.80 ± 0.84	75.44 ± 1.28	0.292
AI (events/h)	6.37 ± 0.76	0.77 ± 0.14	**<0.001**
HI (events/h)	15.69 ± 0.98	1.68 ± 0.23	**<0.001**
AHI (events/h)	22.06 ± 1.25	2.45 ± 0.22	**<0.001**
Total Chol (mmol/L)	5.00 ± 0.10	4.79 ± 0.16	0.275
HDL-C (mmol/L)	1.13 ± 0.03	1.27 ± 0.05	**0.012**
LDL-C (mmol/L)	3.23 ± 0.09	2.98 ± 0.15	0.162
TG (mmol/L)	1.50 ± 0.09	1.33 ± 0.13	0.260
GLU (mmol/L)	5.89 ± 0.13	5.78 ± 0.26	0.716
HbA1C %	5.87 ± 0.10	5.77 ± 0.18	0.620
WBC (10^9^/L)	7.03 ± 0.18	6.53 ± 0.27	0.126
c-Peptide (pmol/L)	3278.44 ± 140.18	2528.19 ± 222.78	**0.006**
Insulin (U/L)	10.10 ± 0.58	7.93 ± 0.76	**0.025**
IGFBP4 (ng/mL)	337.13 ± 14.36	270.72 ± 18.79	**0.006**
FSTL1 (ng/mL)	10245.53 ± 174.94	13783.33 ± 688.69	**<0.001**
TNF-α (pg/ml)	0.52 ± 0.12	0.64 ± 0.31	0.713
Leptin (ng/mL)	12821.71 ± 901.39	11594.01 ± 1678.12	0.522

Data are Mean ± standard error mean (SEM), SBP, systolic blood pressure; DBP, diastolic blood pressure; ESS, Epworth sleepiness scale; AHI, Apnea-hypopnea index; AI, Apnea index; HI, Hypopnea index.

^#^ Indicates values are reported as frequencies. ** P < 0.01; * P < 0.05. Bolded values indicate statistical significance.

### 3.2 Reduced FSTL1 and a possible correlation with OSA severity

**Figure 2:**
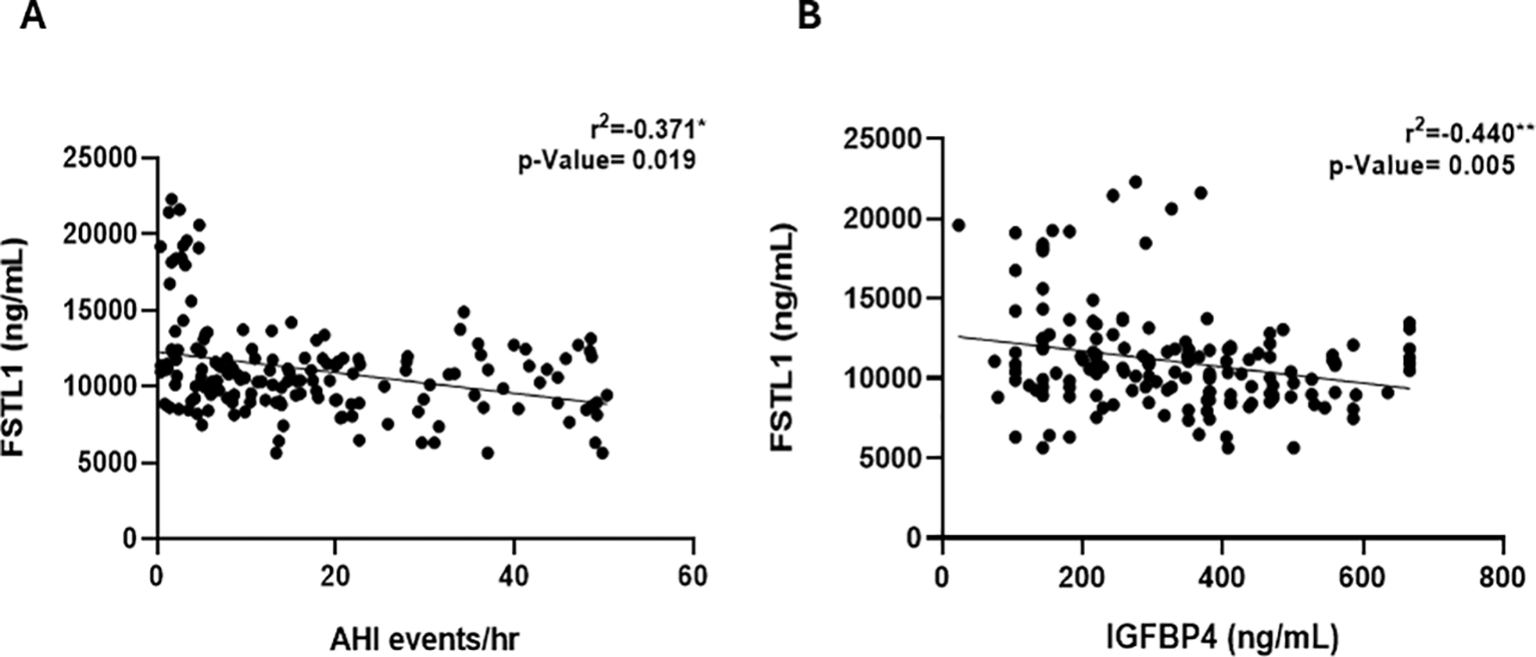
Pearson correlation analysis between circulating FSTL1 and; **(A)** apnea-hypopnea index (AHI) that demonstrates a significant negative correlation (r= -0.371; p-value= 0.019, n=124), **(B)** IGFBP4 displaying a significant negative correlation (r= -0.440; p-value=0.005, n=124). ** P<0.01; * P<0.05.

We quantified baseline circulating FSTL1 levels in all participants and detected a significant decline in FSTL1 levels (10361.74 ± 177.04, p < 0.001) in people with OSA compared to CTRL participants (11675.72 ± 393.20; **Table 1**). We used Pearson’s correlation analysis (**Table 2**) to investigate a potential link between changes in FSTL1 levels and OSA occurrence and/or OSA severity. Notably, FSTL1 demonstrated a significant negative correlation with AHI (r^2^ = -0.195, p = 0.013) in the general population and an even stronger correlation with the OSA group (r^2^ = -0.376, p = 0.019, **Figure 2A**), suggesting a link between FSTL1 and OSA severity. Additionally, the hypoxic marker IGFBP4 was inversely correlated with FSTL1 in OSA patients (r^2^ = -0.440, p = 0.005, **Figure 2B**), suggesting a potential metabolic interaction and a possible compensatory mechanism.

**Table 2:** Pearson correlations with clinical and biochemical parameters.

Variables	All population (n=164)	P value	OSA population (n= 124)	P value
	r		r	
Age (Year)	-0.077	0.329	-0.015	0.866
ESS	-0.186	0.326	0.209	0.391
Body Mass Index (kg/m2)	0.066	0.404	0.093	0.309
AI (events/h)	-0.251	**0.001**	-0.229*	**0.011**
HI (events/h)	-0.255	**0.001**	-0.376	**0.029**
AHI (events/h)	-0.195	**0.013**	-0.371*	**0.019**
Total Cholesterol (mmol/L)	0.025	0.749	0.008	0.933
HDL-C (mmol/L)	0.026	0.740	0.028	0.761
LDL-C (mmol/L)	0.053	0.510	0.045	0.629
TGL (mmol/L)	-0.082	0.303	-0.076	0.407
GLU (mmol/L)	0.103	0.195	0.063	0.494
HBA1C %	0.112	0.163	0.070	0.447
WBC (10^9^/L)	0.061	0.438	0.000	0.998
C-Peptide (pmol/L)	-0.007	0.932	0.041	0.651
Insulin (U/L)	-0.052	0.516	-0.043	0.638
IGFBP4 (ng/mL)	-0.179*	**0.022**	-0.440**	**0.005**
TNF-α (pg/ml)	0.237*	**0.020**	0.088	0.441
Leptin (ng/mL)	0.143	0.071	0.105	0.250

** P < 0.01; * P < 0.05. Bolded values indicate statistical significance.

### 3.3 Resolving OSA is corroborated by elevated circulating FSTL1 levels after surgical intervention

**Figure 3:**
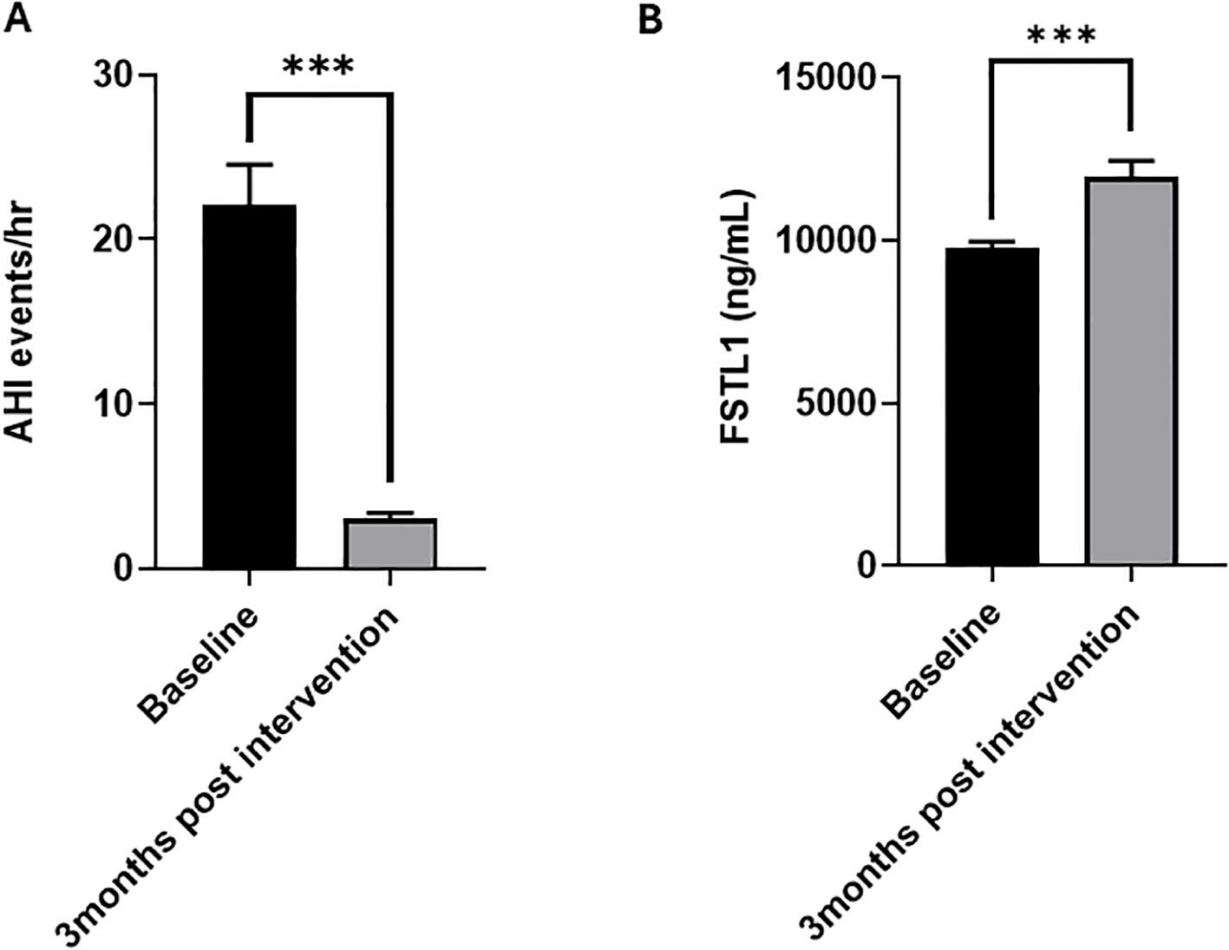
Circulating FSTL1 levels in OSA and non-OSA groups following Multilevel surgery. **(A)** MLS as a corrective intervention for OSA resulted in a significant reduction in AHI (p < 0.001) **(B)** a significant increase in FSTL1 levels after 3 months (11950.22 ± 507.21; 0 <0.001, n=124) compared to baseline (9795.11 ± 250.51).

To further investigate the relationship between OSA and FSTL1 levels, we quantified circulating FSTL1 levels three months after surgery. A significant improvement in OSA indices, particularly in patients with OSA, was observed following surgical intervention. Our data revealed substantial improvements in various sleep indices, including AI (p = 0.003), HI (p < 0.001) and AHI (p < 0.001), confirming an improved airway patency post-surgery. Notably, there is a marked reduction in the AHI three months post-surgery (**Figure 3A**, **Table 3**). This was supported by a significant increase in circulating FSTL1 levels three months after multilevel surgery (MLS) (p < 0.05, **Figure 3B**). The post-surgical rise in FSTL1 suggests that surgical correction of OSA may reverse hypoxia-induced suppression and help restore FSTL1 expression in affected individuals. Additionally, IGFBP4 levels significantly decreased (p = 0.018), aligning with metabolic improvements observed in post-surgical OSA patients.

**Table 3:** Comparison of baseline OSA group to post 3-months intervention.

Variables	Baseline (n=124)	Post 3 months (n=124)	P value
ESS	13.94 ± 1.27	3.33 ± 0.63	**<0.001**
Weight (Kg)	88.92 ± 2.39	88.44 ± 2.31	0.558
Height (cm)	170.99 ± 1.08	171.45 ± 1.10	0.076
Body Mass Index (kg/m2)	30.38 ± 0.67	29.56 ± 0.78	0.127
Pulse	76.17 ± 1.36	75.12 ± 1.35	0.443
SBP (mmHg)	126.73 ± 1.94	126.21 ± 1.65	0.769
DBP (mmHg)	74.06 ± 1.38	75.60 ± 1.21	0.184
AI (events/h)	5.75 ± 1.60	0.86 ± 0.17	**0.003**
HI (events/h)	16.36 ± 1.88	4.33 ± 0.63	**<0.001**
AHI (events/h)	22.11 ± 2.45	3.05 ± 0.35	**<0.001**
Total Cholesterol (mmol/L)	5.07 ± 0.16	4.95 ± 0.15	0.174
HDL-C (mmol/L)	1.08 ± 0.03	1.11 ± 0.04	0.331
LDL-C (mmol/L)	3.30 ± 0.15	3.18 ± 0.15	0.123
TGL (mmol/L)	1.56 ± 0.14	1.49 ± 0.11	0.542
Glucose (mmol/L)	5.85 ± 0.16	5.76 ± 0.16	0.412
HBA1C %	5.77 ± 0.12	5.75 ± 0.11	0.827
C-Peptide (pmol/L)	3460.48 ± 210.78	3425.63 ± 212.63	0.829
Insulin (U/L)	9.95 ± 0.85	10.73 ± 1.03	0.330
IGFBP4 (ng/mL)	408.81 ± 19.94	337.18 ± 23.72	**0.018**
FSTL1 (ng/mL)	9795.11 ± 250.51	11950.22 ± 507.21	**<0.001**
TNF-α (pg/mL)	0.31 ± 0.09	0.34 ± 0.13	0.864
Leptin (ng/mL)	12006.69 ± 1279.70	10925.86 ± 1047.14	0.193

Data are Mean ± standard error mean (SEM), SBP, systolic blood pressure; DBP, diastolic blood pressure; ESS, Epworth sleepiness scale; AHI, Apnea-hypopnea index; AI, Apnea index; HI, Hypopnea index. ** P < 0.01; * P < 0.05 Bolded values indicate statistical significance.

### 3.4 FSTL1 and the potential to predict OSA

We performed stepwise linear regression analysis (**Table 4**) using age, BMI, gender, ESS, AHI, and IGFBP4 levels as predictors of FSTL1 levels in the total study population and the OSA group. Our analysis presents AHI index as the strongest negative predictor of FSTL1 in the total population (p = 0.002) and in people with OSA (p = 0.001), which highlights the inverse relationship between OSA severity and FSTL1. Additionally, the analysis shows that IGFBP4 is a significant independent predictor, correlated with FSTL1 in the total population (p = 0.011) and in people with OSA (p = 0.005), suggesting a metabolic role in FSTL1 regulation.

**Table 4:** Multiple Stepwise Linear Regression Analysis for FSTL1 Predictors.

Variables	All Population (n=164)		OSA Population (n=124)	
	β	p-value	β	p-value
AHI (events/h)	- 0.252	**0.002**	- 0.284	**0.001**
IGFBP4 (ng/ml)	- 0.200	**0.011**	- 0.221	**0.005**

Variables used in linear regression model; age, BMI, gender, ESS, AHI, and IGFBP4. ** P < 0.01; * P < 0.05. Bolded values indicate statistical significance.

### 3.5 Receiver operating characteristic curve analysis of FSTL1 for OSA prediction

**Figure 4:**
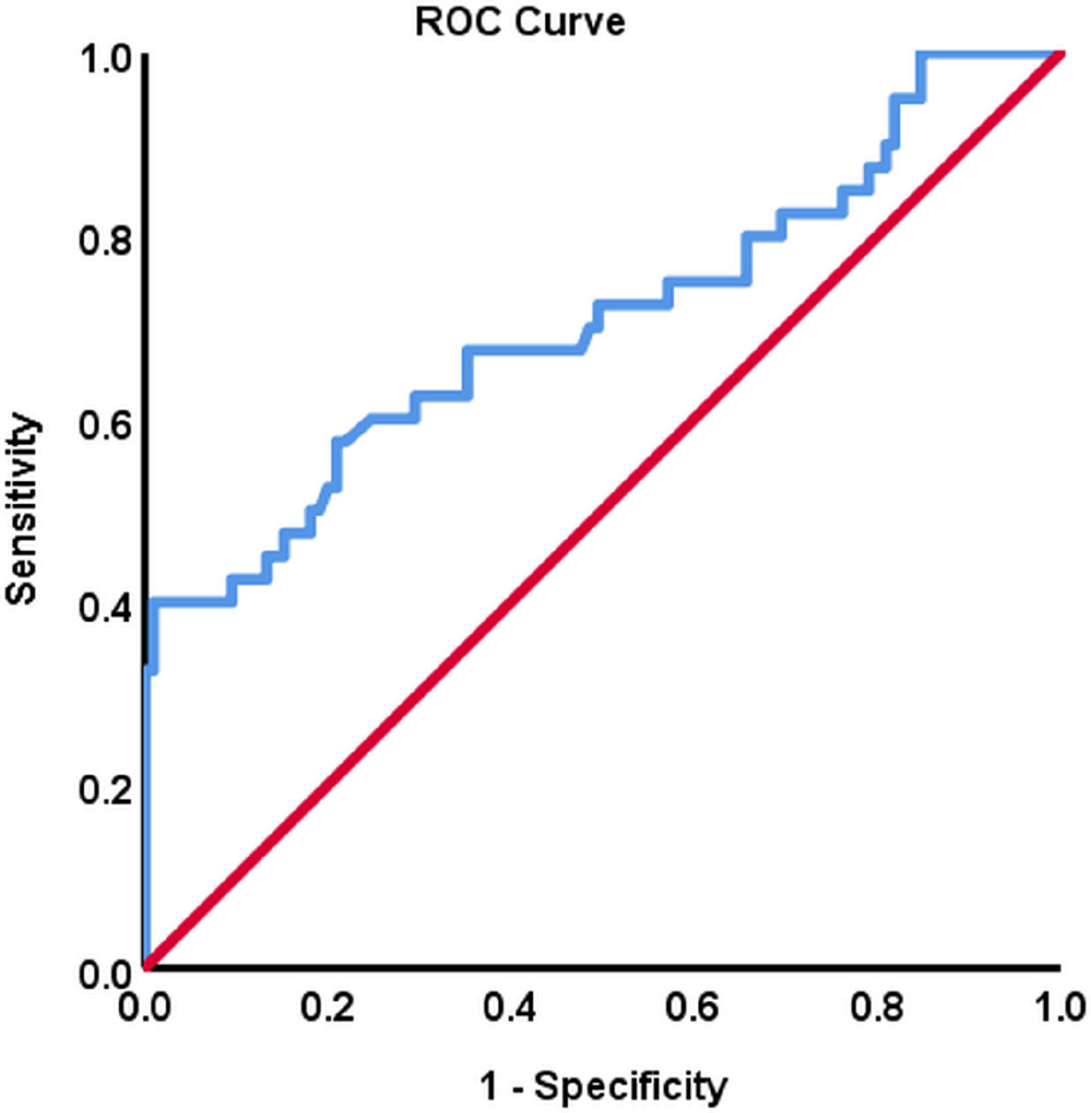
ROC curve analysis of FSTL1 for OSA prediction to identify the cut-off value for FSTL1 for OSA. The area under the curve (AUC) for FSTL1 0.73 (95% CI: 0.64–0.83, p < 0.001, n=164).

We performed ROC curve analysis to evaluate FSTL1 as a predictive biomarker for OSA (moderate to severe cases) and to identify the optimal FSTL1 cut-off value (**Figure 4**). Our analysis demonstrated moderate diagnostic accuracy for FSTL1 in predicting OSA (AUC 0.73, 95% CI: 0.64–0.83, p < 0.001; 8819.09; sensitivity of 86.4%, specificity of 76.2%). The optimal Youden cut-off value for predicting OSA using FSTL1 was 8819.09 ng/mL, with an 86.4% sensitivity and 76.2% specificity, highlighting the potential utility of FSTL1 as a biomarker for OSA diagnosis.

## 4 Discussion

This report demonstrates a significant reduction in baseline circulating FSTL1 levels in individuals with OSA compared with those without OSA. This decrease was negatively correlated with OSA severity. Following MLS intervention, FSTL1 levels increased significantly, accompanied by notable improvements in key clinical parameters and sleep indices. ROC curve analysis further highlighted the sensitivity and specificity of FSTL1 as a potential biomarker linked to OSA. Together, these findings support FSTL1 as a promising novel candidate that can aid in diagnosing OSA alongside traditional methods and in monitoring treatment outcomes.

Follistatin-like 1 is one of five secreted glycoproteins from the follistatin-like proteins family (14). Members of the FSTL family are widely expressed, exhibiting a range of paracrine and autocrine activities that depend on their site of expression and the physiological or pathological condition (14). FSTL1 is primarily expressed in the heart, lungs, and white adipose tissue and plays essential roles in regulating oxidative stress and inflammation, in addition to its cardiovascular protective effects (14). At normal physiological conditions, FSTL1 is activated by cardiac stress to alleviate hypertrophy and systolic dysfunction (15, 16). FSTL1 has a broad range of physiological functions, including regulating endothelial cells’ activity, promoting revascularization and modulating metabolic pathways in cardiovascular disorders (17, 18). In a condition characterized by chronic intermittent hypoxia, such as OSA, FSTL1 levels decline severely in lung cancer tissue, which aggravates oxidative stress and inflammatory responses (11). Nonetheless, restoration of FSTL1 expression or FSTL1 overexpression normalized oxidative and inflammatory pathways by mitigating the damage caused by chronic intermittent hypoxia (11). The simultaneous ability of FSTL1 to promote cell survival and inhibit inflammatory signaling highlights its potential importance in OSA-related disorders. This implicates FSTL1 as a viable therapeutic target that might help alleviate OSA-associated problems (19).

Previous studies have reported that FSTL1 plays a dynamic role in response to hypoxia, functioning as a hypoxia-responsive protein in some contexts while protecting against hypoxic damage in others (20, 21). Our data show that FSTL1 levels are significantly reduced in OSA patients compared to non-OSA controls, supporting the hypothesis that FSTL1 might be linked to the pathophysiological mechanisms of OSA, which might involve inflammation and oxidative stress. This finding aligns with reports showing FSTL1 suppression in response to prolonged hypoxia, which has been attributed to a potential contribution to disease progression (20). The observed negative correlation between FSTL1 and AHI further suggests a link between OSA severity and FSTL1 suppression.

The presence of an inverse relationship between FSTL1 and hypoxia was shown by Qi et. al, whereby FSTL1-deficient mice displayed increased levels of IH-induced tumor metastasis (11). The rise in pathological markers was attenuated by FSTL1 overexpression, which normalized oxidative stress and inflammatory pathways and reduced tumor cell migration (11). This comes in agreement with our data emphasizing that OSA severity, which is mediated through IH, may inhibit FSTL1 expression, hence reducing its beneficial activities in mitigating hypoxia-driven cellular damage and inflammation. To our knowledge, our study is the first to show the FSTL1-OSA link in human OSA population. Nonetheless, additional studies are needed to explore this relationship further and identify the molecular determinants involved in this process. Interestingly, the FSTL1-OSA link is further confirmed by our pre- and post-MLS quantification of FSTL1 levels. A key finding of our study is a significant increase in FSTL1 levels after 3 months of MLS, suggesting that surgical intervention may facilitate the restoration of FSTL1 expression. This aligns with previous studies indicating that OSA treatments, such as MLS, can mitigate inflammatory responses (22), potentially influencing FSTL1 regulation. Beyond reflecting short-term treatment response, these findings suggest that FSTL1 could serve as a dynamic biomarker for monitoring longitudinal disease trajectories. Serial measurement of FSTL1 beyond the 3 months may reveal whether its normalization is sustained, whether fluctuations correspond to OSA relapse or persistence, and if it carries prognostic value for long-term cardiovascular or metabolic outcomes. Such insights would broaden the clinical utility of FSTL1 beyond a diagnostic and short-term monitoring biomarker to potentially serve as a predictive tool for long-term disease stability and recurrence risk.

Furthermore, our data show a significant decrease in IGFBP4 levels post-surgery (p = 0.018), confirming a link to OSA (23). In the context of OSA, both FSTL1 and IGFBP4 may be relevant due to their involvement in inflammation, oxidative stress, and tissue remodeling (24–26). Obstructive Sleep Apnea is characterized by IH and systemic inflammation, which would impact the expression and function of proteins involved in these pathways. FSTL1 is known to be regulated by hypoxia and to influence inflammatory and fibrotic responses in several tissues (24, 27), and the role of IGFBP4 in OSA has been reported (23). Taken together, these proteins could have a role in the tissue remodeling and inflammatory processes associated with OSA; however, their relationship is indirect and might be context dependent. While our findings suggest an OSA-related link to both FSTL1 and IGFBP4, further research, particularly mechanistic and longitudinal studies, is needed to confirm whether a direct association or functional interaction exists between these proteins in OSA or other disease models.

Regression analysis identified AHI and IGFBP4 as independent predictors of FSTL1 levels, reinforcing the notion that OSA severity and metabolic alterations directly influence FSTL1 levels. This highlights FSTL1 as a potential biomarker for OSA diagnosis and a possible indicator of treatment efficacy. The ROC curve analysis further supports the value of FSTL1 in an OSA-linked mechanism, demonstrating moderate diagnostic accuracy (AUC = 0.73) with high sensitivity (86.4%) and specificity (76.2%). While these findings are encouraging, the moderate accuracy suggests that FSTL1 may be most valuable when used in combination with other established or emerging biomarkers—such as inflammatory cytokines, hypoxia-related proteins, or metabolic regulators, rather than as a standalone tool. Such composite biomarker panels could improve diagnostic precision, enhance patient stratification, and strengthen monitoring of treatment responsiveness, thereby complementing traditional sleep study assessments. If validated in larger and multi-center cohorts, FSTL1 could complement PSG by serving as a rapid and preliminary screening tool and a monitoring biomarker for treatment response, thereby enhancing clinical applicability and aligning with efforts to improve patient care.

Despite these promising findings, we acknowledge several limitations. First, our study had a moderate sample size and relied on only two-point FSTL1 measurements. Second, it was conducted at a single center in Kuwait, which may limit the external validity and generalizability of the findings to other populations. Third, the follow-up period was relatively short (3 months post-surgery) and thus, the long-term stability and trajectory of FSTL1 dynamics remain uncertain. Finally, while our study demonstrates associations among OSA severity, hypoxia, inflammation, and FSTL1 levels, it does not provide mechanistic insights into the causal pathways underlying these relationships. No multiple-testing correction was applied; future studies will incorporate FDR adjustment to reduce false positives. Future multicenter, longitudinal studies with larger cohorts and mechanistic exploration are warranted to validate and expand on our findings.

## 5 Conclusions

This study demonstrates that OSA is associated with altered metabolic and inflammatory pathways, with FSTL1 levels markedly reduced in affected individuals. MLS intervention restored FSTL1 levels, reinforcing its potential as a hypoxia-related diagnostic biomarker and an indicator of treatment efficacy. Regression analysis further identified predictors of FSTL1, underscoring its link to OSA severity and metabolic dysfunction. While these findings suggest FSTL1 as a promising biomarker for disease progression and therapeutic response, further studies are warranted to validate its clinical utility and explore its role in OSA-related cardiometabolic complications.

## Data Availability

The original contributions presented in the study are included in the article/**Supplementary Material**. Further inquiries can be directed to the corresponding author/s.

## Supplementary material

Supplementary Table 1 Clinical and Biochemical characteristics of participants by BMIData are Mean ± standard error mean (SEM), ESS: Epworth sleepiness scale; AHI: Apnea-hypopnea index; AI: Apnea index; HI: Hypopnea index. ^#^ Indicates values are reported as percent. ** P < 0.01; * P < 0.05.
