# Supplementary material for: Follistatin-like 1: a novel biomarker with a potential link to obstructive sleep apnea severity and treatment efficacy
Source: Front Endocrinol (Lausanne). 2025 Dec 11;16:1690401. doi: 10.3389/fendo.2025.1690401 (PMC12738361; doi:10.3389/fendo.2025.1690401)
Supplement: Supplementary Table 1 — Clinical and Biochemical characteristics of participants by BMIData are Mean ± standard error mean (SEM), ESS: Epworth sleepiness scale; AHI: Apnea-hypopnea index; AI: Apnea index; HI: Hypopnea index. # Indicates values are reported as percent. ** P < 0.01; * P < 0.05. [file Table1.docx]

Supplementary Table1: Clinical and Biochemical characteristics of participants by BMI

| Variables | Non-Obese (n=25) mean ± SEM | Obese (n=139)  mean ± SEM | p-value |
| --- | --- | --- | --- |
| Age (Years) | 44.72 ± 2.35 | 43.68 ± 0.94 | 0.685 |
| Gender (m%/f%) ^#^ | 21/4 (84/16) | 117/22 (84/16) | 0.983 |
| ESS | 5.63 ± 2.24 | 11.45 ± 1.44 | 0.047 |
| Weight (Kg) | 68.46 ± 1.37 | 92.87 ± 1.41 | <0.001 |
| Height (cm) | 170.34 ± 1.97 | 171.32 ± 0.75 | 0.646 |
| Body Mass Index (kg/m^2^) | 23.52 ± 0.25 | 31.52 ± 0.38 | <0.001 |
| Pulse | 75.09 ± 2.56 | 74.68 ± 0.93 | 0.883 |
| SBP (mmHg) | 120.57 ± 3.06 | 126.46 ± 1.06 | 0.080 |
| DBP (mmHg) | 70.96 ± 1.75 | 74.77 ± 0.77 | 0.055 |
| AI (events/h) | 2.94 ± 0.67 | 5.38 ± 0.70 | 0.014 |
| HI (events/h) | 6.05 ± 1.74 | 13.40 ± 0.96 | 0.001 |
| AHI (events/h) | 8.99 ± 2.17 | 18.77 ± 1.26 | <0.001 |
| Total Cholesterol (mmol/l) | 4.87 ± 0.23 | 4.96 ± 0.09 | 0.707 |
| HDL-C (mmol/l) | 1.27 ± 0.07 | 1.14 ± 0.03 | 0.090 |
| LDL-C (mmol/l) | 3.01 ± 0.21 | 3.19 ± 0.08 | 0.419 |
| Triglycerides (mmol/l) | 1.23 ± 0.21 | 1.50 ± 0.08 | 0.242 |
| Glucose (mmol/l) | 5.66 ± 0.30 | 5.90 ± 0.13 | 0.471 |
| HBA1C % | 5.57 ± 0.12 | 5.89 ± 0.10 | 0.045 |
| Whole Blood Counts (10^9^/L) | 6.17 ± 0.42 | 7.04 ± 0.16 | 0.063 |
| C-Peptide (pmol/L) | 1961.75 ± 205.25 | 3295.28 ± 131.22 | <0.001 |
| Insulin (U/L) | 5.10 ± 0.62 | 10.35 ± 0.53 | <0.001 |
| IGFBP4 **(ng/mL)** | 247.29 ± 27.54 | 334.18 ± 12.95 | 0.007 |
| FSTL1 **(ng/mL)** | 11458.37 ± 618.22 | 11045.46 ± 265.51 | 0.544 |
| TNF-α (pg/mL) | 0.28 ± 0.27 | 0.57 ± 0.12 | 0.333 |
| Leptin (ng/mL) | 6167.37 ± 2423.67 | 13643.94 ± 795.98 | 0.007 |

Data are Mean ± standard error mean (SEM), ESS: Epworth sleepiness scale; AHI: Apnea-hypopnea index; AI: Apnea index; HI: Hypopnea index. ^#^ Indicates values are reported as percent. ** P < 0.01; * P < 0.05.
